# Primary headache disorders among the adult population of Mongolia: prevalences and associations from a population-based survey

**DOI:** 10.1186/s10194-019-1061-5

**Published:** 2019-12-16

**Authors:** Otgonbayar Luvsannorov, Byambasuren Tsenddorj, Dorjkhand Baldorj, Selenge Enkhtuya, Delgermaa Purev, Hallie Thomas, Timothy J. Steiner

**Affiliations:** 1 Department of Neurology, Mongolian National University of Medical Sciences, Ulaanbaatar, Mongolia; 2 Department of Neurology, Ach Medical University, Ulaanbaatar, Mongolia; 3 Division of Neurology, Мungunguur Clinical Hospital, Ulaanbaatar, Mongolia; 4 Department of Neuromedicine and Movement Science, Norwegian University of Science and Technology, Edvard Griegs gate, Trondheim, Norway; 5 Division of Brain Sciences, Imperial College London, London, UK

**Keywords:** Headache, Migraine, Tension type headache, Medication-overuse headache, Prevalence, Associations, Population-based study, Mongolia, Global Campaign against Headache

## Abstract

**Background:**

In the ongoing Global Campaign endeavour to improve knowledge and awareness of headache prevalence worldwide, Mongolia is a country of interest. It sits between Russia and China, in which prevalence is, respectively, much higher and much lower than the estimated global mean. We conducted a population-based study in Mongolia both to add to knowledge and to inform local health policy.

**Methods:**

Using standardized methodology with cluster random sampling, we selected Mongolian adults (aged 18–65 years) from five regions reflecting the country’s diversities. They were interviewed by trained researchers, cold-calling at their homes, using the Headache-Attributed Restriction, Disability, Social Handicap and Impaired Participation (HARDSHIP) structured questionnaire following pilot-testing. ICHD-3 beta diagnostic criteria were applied.

**Results:**

*N* = 2043 (mean age 38.0 [±13.4] years, 40% urban-dwelling and 60% rural), with a non-participation proportion of 1.7%. Males were somewhat underrepresented, for which corrections were made. The crude 1-year prevalence of any headache was 66.1% (95% CI: 64.0–68.2%), with a strong female preponderance (OR: 2.2; *p* < 0.0001). Age- and gender-adjusted prevalences were: migraine 23.1% (for females, OR = 2.2; p < 0.0001); tension-type headache (TTH) 29.1% (no gender difference); probable medication-overuse headache (pMOH) 5.7% (trending towards higher in females); other headache on ≥15 days/month 5.0% (for females, OR = 2.2; *p* = 0.0008). Unclassified cases were only 35 (1.7%). Any headache yesterday was reported by 410 (20.1%; for females, OR = 2.4; p < 0.0001). Only pMOH showed a strong association with age, peaking in middle years with a 5-fold increase in prevalence. Migraine showed a consistent association with educational level, while pMOH showed the reverse, and was also more common among other groups than among participants who were single (never married). Migraine was less common among rural participants than urban (OR: 0.80; *p* = 0.0326), while pMOH again showed the reverse (OR: 2.4; *p* < 0.0001). Finally, pMOH (but not migraine or TTH) was significantly associated with obesity (OR: 1.8; *p* = 0.0214).

**Conclusion:**

Headache disorders are common in Mongolia, with, most notably, a very high prevalence of headache on ≥15 days/month corroborated by the high prevalence of headache yesterday. The picture is very like that in Russia, and dissimilar to China. There are messages for national health policy.

## Introduction

Headache disorders are now acknowledged as the most prevalent cause of public ill health, affecting people in all countries, and as the second highest cause of disability worldwide [1–3]. This realization has come, in the main, from the multiple iterations of the Global Burden of Disease (GBD) study since the year 2000 [2, 4]. GBD2010 found tension-type headache (TTH) and migraine to be the 2nd and 3rd most prevalent disorders in the world (only dental caries was more common) [5]. GBD2016 and GBD2017 established their high ranking (collectively second) among all causes of years lived with disability (YLDs) [2–4, 6, 7].

GBD has been increasingly well informed during these years, notably by the population-based studies supported in countries around the world by *Lifting The Burden* (LTB) [8–12]. Nevertheless, knowledge of the prevalence of headache disorders, on which GBD depends, remains incomplete [1, 13]. Furthermore, while LTB’s studies all use similar, standardized methodology developed for the purpose [14, 15], regional variations have been noted in headache prevalence. Of particular interest in this context are the data from Russia [16] and China [17], countries with a common border, in both of which LTB has supported studies. These had highly disparate findings: whereas prevalences were high in Russia for migraine (20.8%), TTH (30.8%) and, especially, headache on ≥15 days/month (10.5%), in China these were notably below global averages (9.3%, 10.8% and 1.0% respectively – even though headache disorders were still a substantial cause of public ill health here). Russia in this respect appears not so different from the Republic of Georgia [18] and Lithuania [19], also countries of the former USSR. China appears to be matched by Japan [20] and Taiwan [21], suggesting that, in these countries of the Far East, cultural and/or genetic distinctions influence headache prevalence [22] (although how these influences might work is unknown).

No epidemiological data on headache are available from Mongolia, but this country is of considerable interest here. Sitting between Russia to the north and China to the south, it is nonetheless ethnically different from both while, historically, culturally influenced by both. Its population is young (average age 27.5 years, with almost 60% under 30 [23]), but neither large (currently estimated at 3.17 million) nor diverse: about 95% are ethnic Mongols, with Turkic people accounting for 4.5% [23]. Nevertheless, the country displays some unique sociodemographic characteristics. Large parts of it, including Ulaanbaatar, the Capital city, lie at altitudes between 1000 and 1500 m. There is an urban/rural population divide in the ratio approximately of 40% to 60%. Rural areas include the steppes and Gobi Desert, and rural life may not be easy: unique to Mongolia is the *dzud*, a summer drought followed by a severe winter, causing serious loss of livestock and characterized as a natural disaster. While Ulaanbaatar is the only conurbation with a population > 100,000 [23], urbanization – by migration from the steppes into Ulaanbaatar – has been rapid and is expanding [24]. Increasingly this is into the so-called *ger* areas, unplanned neighbourhoods which now house some 60% of Ulaanbaatar’s population while often lacking sanitation, water, electricity and heating. Only 10% of these areas have paved roads, challenging public transportation, health services and access to schools [24]. Household incomes in the ger areas are estimated to be half those elsewhere in the city [24], but there are many informal inhabitants whose residence is not legally registered.

Accordingly, continuing LTB’s series of studies, we undertook a nationwide population-based survey in Mongolia. We focused on the headache disorders of public-health importance (migraine, TTH, medication-overuse headache [MOH] and other headache occurring on ≥15 days/month), the purposes being two-fold: to add to the global map of headache, and, more importantly, to inform public-health policy in the country. Here we describe the 1-year prevalence of these headache disorders in the adult population, and present analyses of associations with demographic variables. Subsequent papers will report headache-attributed burden.

## Materials and methods

### Ethics

The Ethics Committee of Research of the Mongolian National University of Medical Sciences (MNUMS) approved the study protocol. Informed written (signed) consent was obtained from all participants.

Data were handled with due regard to data protection legislation.

### Study design

The study was a cross-sectional, population-based survey among randomly-selected Mongolian adults (aged 18–65 years) conducted in accordance with published methodology [14, 15].

The study design incorporated a pilot study (results not presented) to ensure the methods worked before commitment of resources to the main study.

Data were collected during August to November 2017.

### Sampling and selection

**Fig. 1:**
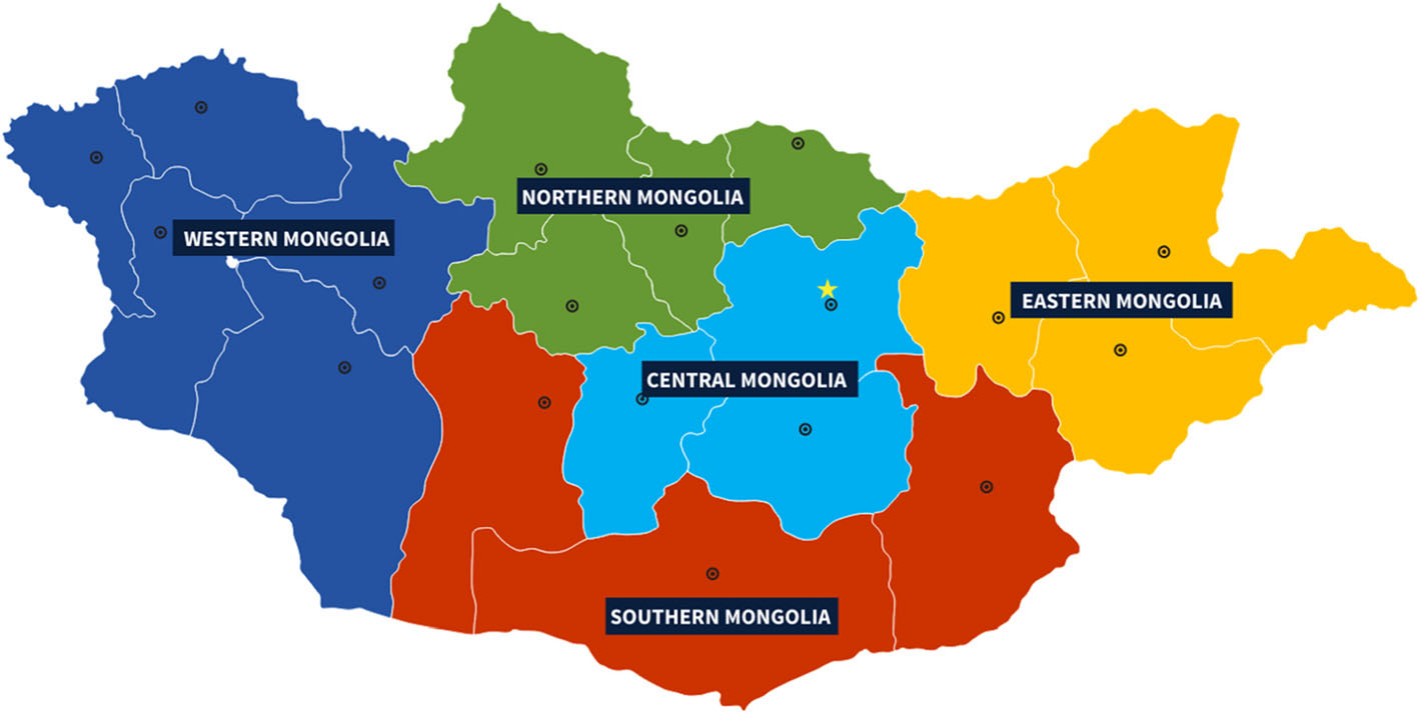
Mongolia’s regions (Ulaanbaatar, in Central region, marked by star)

Through multistage randomized cluster-sampling, participants representative of the adult general population of the country were identified. The survey included five areas to reflect the country’s geographical diversity and provide an appropriate mix of urban and rural participants (matching the country’s population divide). Numbers recruited in each area reflected their population sizes: 40% from Ulaanbaatar (urban, and at 1300 m) and 15% from each of four aimags (one selected randomly from each of Mongolia’s other four geographical regions (Fig. 1), all rural): Zavkhan (Western region; 2000–2200 m), Arkhangai (Khangai region; 1000–1300 m), Umnugovi (Gobi region; 1300–1600 m) and Dornod (Eastern region; 800–900 m).

In Ulaanbaatar, we surveyed four randomly selected districts, including the ger areas. In each aimag, we convenience-selected the village closest to the point of arrival from Ulaanbaatar. We selected streets randomly, and each dwelling consecutively in these streets, until requisite numbers were achieved.

Access to each individual was by door-to-door cold-calling at selected households, with random selection of one adult member of each biologically unrelated family (as the final sampling unit) within each household. This selected respondent was included in the sample, subject to their consent; in the event of refusal, replacement from the same household was not permitted. Nevertheless, some bias arose here (for which statistical correction was later made) because male workers were commonly absent for extended periods, reducing their probability of selection.

### Interviews

In face-to-face interviews, eight trained neurologist interviewers employed the Headache-Attributed Restriction, Disability, Social Handicap and Impaired Participation (HARDSHIP) structured questionnaire [14], culturally adapted and translated into Mongolian language in accordance with LTB’s translation protocol for lay documents [25].

HARDSHIP has a modular format [14]. For this survey it included, for all participants, demographic enquiry and a headache screening question (“Have you had headache during the last year?”). Those who answered “no” to this question were classified as headache-free. For all those reporting headache, diagnostic questions followed, based on the International Classification of Headache Disorders (ICHD-3 beta) [26], along with enquiries into burden. Any participant reporting more than one headache type was asked to focus only on the one that was subjectively the most bothersome for purposes of description, diagnosis and prevalence counting [15]. We also asked if headache had occurred on the day preceding the enquiry (“headache yesterday”). Finally, we enquired into body weight and height.

### Data management

We entered data into a secure database. All were independently entered twice, with reconciliation and correction of errors by reference to the original questionnaires.

### Statistics and analyses

We accepted published power calculations recommending *N* = 2000 as the best compromise between statistical power and conservation of resources [15].

We categorized age in five groups (following those used in the national census [27]: 18–24, 25–34, 35–44, 45–54 and 55–65 years). We categorized education into four levels (elementary, secondary, college and university), and combined the last two for association analysis. We categorized marital status as single (never married), married (and living with marital partner), widowed, or divorced/separated, and again combined the last two for association analysis. We categorized employment as employed, unemployed (including housewives who were not otherwise employed), student or retired, once more combining the last two for association analysis. Although we did not measure the altitude of each household, we had altitude data for each of the survey areas (see “Sampling and selection” above), according to which we categorized this variable as < 1000 m, 1000–2000 m or > 2000 m. We took waist measurement (ignoring those known to be pregnant) and recorded self-reported height and weight, calculating body-mass index (BMI) as (weight in Kg)/(height in m)^2^.

Diagnoses were made not by the interviewers but by computerized algorithm [13] from the recorded survey responses. Participants reporting headache on ≥15 days/month were first separated, and described as a distinct group, with those also reporting regular use of acute headache medication on > 15 days/month (triptans and combination analgesics being used rarely in Mongolia) considered to have probable MOH (pMOH). To all others, the algorithm applied ICHD-3 beta diagnostic criteria [26] in the order: migraine, TTH, probable migraine, probable TTH [15]. Cases of migraine and probable migraine, and of TTH and probable TTH, were then combined for prevalence estimation and further analyses [15]. Remaining cases were unclassified.

Statistical analyses were performed using Excel or SPSS v25. We estimated crude 1-year prevalence for all headache, migraine and TTH, and point prevalence for pMOH and other headache on ≥15 days/month. One-day prevalence of headache was represented by headache yesterday. We adjusted these for age and gender. We used proportions, 95% confidence intervals (CIs), medians, means and standard deviations (SDs) to summarize the distributions of variables, and chi-squared and Student’s t-test for significance of differences. We calculated odds ratios (ORs) to test for associations in bivariate analysis. We set the level of significance at 5%.

## Results

A total of 2379 households were visited. Those not responding (*n* = 299) were excluded, since it could not be ascertained whether any occupants were eligible. There were 36/2080 refusals (non-participation proportion 1.7%). The survey thus recruited 2043 participants, mean age 38.0 [±13.4] years, 817 [40.0%] from Ulaanbaatar and 303–311 (total, 1226: 60.0%] from the four aimags. Table 1 shows that males were somewhat underrepresented. The distributions of age in the participating sample and total population of Mongolia were similar, although differing statistically (chi-squared = 40.142; df = 4; p < 0.0001). Habitation in the sample matched that of the population.

**Table 1:** Sociodemographic characteristics of the participating sample (*N* = 2043) and national population

Variable	Sample *n* (%)	National population^a^ %
Habitation		
Urban	817 (40.0)	40^b^
Rural	1226 (60.0)	60^b^
Gender		
Male	813 (39.8)	48.5
Female	1230 (60.2)	51.5
Age (years)		
18–24	383 (18.8)	16.9
25–34	567 (27.8)	30.0
35–44	416 (20.4)	23.7
45–54	369 (18.1)	18.1
55–65	306 (15.0)	11.4
Education		
Elementary	82 (4.0)	28.4
Secondary	690 (33.8)	34.6
College	291 (14.2)	12.7
University	980 (48.0)	19.8
Marital status		
Single (never marred)	481 (23.5)	32.3
Married	1341 (65.6)	57.1
Widowed	140 (6.9)	29.2
Divorced or separated	81 (4.0)	not reported
Employment		
Employed	1182 (57.9)	47.4
Unemployed or housewife	350 (17.1)	15.3
Student	274 (13.4)	not reported
Retired	237 (11.6)	10.0

^a^From [27]. ^b^Estimated according to [24]

### Headache prevalence

Of the 2043 participants, 1351 reported headache in the last year. The crude 1-year prevalence of any headache in the study population was 66.1% (95% CI: 64.0–68.2%), with a strong female preponderance (73.3% [70.7–75.7%] versus 55.4% [51.9–58.7%] in males; OR: 2.2; *p* < 0.0001). The following prevalences are shown in Table 2.

**Table 2:** Observed 1-year prevalence (% [95% confidence intervals]) by gender and age, overall and by headache type, and adjusted values for gender and age

	Migraine (*n* = 492)	TTH (*n* = 596)	pMOH (*n* = 120)	Other headache on ≥15d/m (*n* = 108)	Any headache yesterday (*n* = 410)
All (*N* = 2043)	24.1 [22.3–26.0]	29.2 [27.2–31.2]	5.9 [4.9–6.9]	5.3 [4.3–6.3]	20.1 [18.4–21.9]
Gender					
Male (*n* = 813)	16.0 [13.5–18.5]	29.4 [26.3–32.5]	4.7 [3.3–6.2]	3.2 [2.0–4.4]	12.2 [10.0–14.5]
Female (*n* = 1230)	29.4 [26.9–32.0]	29.0 [26.5–31.5]	6.7 [5.3–8.1]	6.7 [5.3–8.1]	25.3 [22.9–27.7]
Age (yr)					
18–24 (*n* = 383)	25.6 [21.2–30.0]	29.8 [25.2–34.4]	1.8 [0.5–3.1]	3.9 [2.0–5.8]	17.2 [13.4–21.0]
25–34 (*n* = 567)	25.4 [21.8–29.0]	33.0 [29.1–36.9]	3.0 [1.6–4.4]	6.0 [4.1–8.0]	18.2 [15.0–21.4]
35–44 (*n* = 416)	24.0 [19.9–28.1]	27.6 [23.3–31.9]	9.1 [6.3–11.9]	5.0 [2.9–7.1]	21.9 [17.9–25.9]
45–54 (*n* = 369)	24.4 [20.0–28.8]	27.6 [23.0–32.2]	8.9 [6.0–11.9]	5.7 [3.3–8.1]	24.1 [19.7–28.5]
55–65 (*n* = 306)	19.6 [15.2–24.1]	25.2 [20.3–30.1]	8.2 [5.1–11.3]	5.6 [3.0–8.2]	19.9 [15.4–24.4]
Adjusted for gender and age	23.1%	29.1%	5.7%	5.0%	19.0%

*TTH* Tension-type headache, *pMOH* Probable medication-overuse headache, *d/m* days/month.

**Fig. 2:**
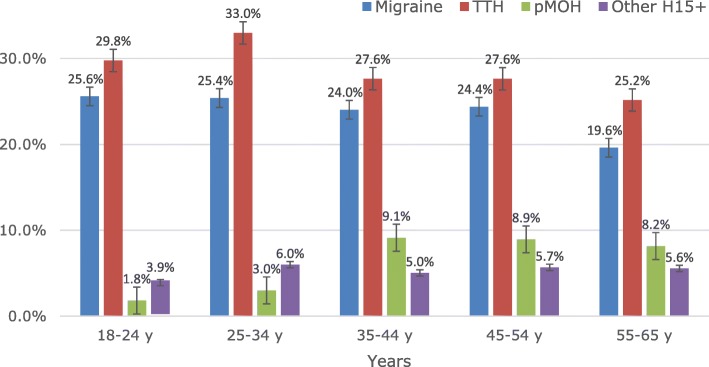
Age related prevalence of each headache type (TTH: tension-type headache; pMOH: probable medication-overuse headache; Other H15+: other headache on ≥15 days/month)

The crude 1-year prevalence of migraine was 24.1% (*n* = 492), 8.7% (*n* = 178) definite and 15.4% (*n* = 314) probable. Prevalence was substantially higher among females (29.4%) than males (16.0%; OR: 2.2; p < 0.0001). Prevalence of migraine showed little variation with age until 55–65 years, when it decreased from about 25% to 19.6% (Table 2; Fig. 2). The age- and gender-adjusted prevalence of migraine was 23.1%.

The crude 1-year prevalence of TTH was 29.2% (*n* = 596), 20.7% (*n* = 423) definite and 8.5% (*n* = 173) probable. Prevalence was similar among males (29.4%) and females (29.0%), with some increase with age to a peak of 33.0% at 25–34 years then a decline to 25.2% (Table 2; Fig. 2). The age- and gender-adjusted prevalence of TTH was 29.1%.

The crude prevalence of pMOH was 5.9% (*n* = 120). There was a trend towards higher prevalence among females (6.7%) than males (4.7%; *p* = 0.0621), and a strong age relationship (prevalence increasing from 1.8% in those under 25 years to about 9% during ages 35–54 years (Table 2; Fig. 2). The age- and gender-adjusted prevalence of pMOH was 5.7%.

The crude prevalence of other headache on ≥15 days/month was 5.3% (n = 108), higher among females (6.7%) than males (3.2%; OR: 2.2; p = 0.0008). Prevalence was lowest in those aged under 25 years (3.9%) but variations after age 25 (5.0–6.0%) were insignificant (Table 2; Fig. 2). The age- and gender-adjusted prevalence of other headache on ≥15 days/month was 5.0%.

Unclassified cases (*n* = 35) were only 1.7%.

Headache yesterday was reported by 410 participants (20.1%), females (25.3%) more than males (12.2%; OR: 2.4; *p* < 0.0001).

### Associations

Associations with age and gender have been noted. Others are illustrated in Table 3.

**Table 3:** Bivariate analysis of associations with the principal headache types

	Migraine			Tension-type headache			pMOH		
	Odds	OR [95% CI]	*p*	Odds	OR [95% CI]	*p*	Odds	OR [95% CI]	*p*
Habitation									
Urban	0.36	reference	–	0.45	reference	–	0.05	reference	–
Rural	0.29	0.80 [0.65–0.98]	0.0326	0.39	0.85 [0.70–1.03]	0.0981	0.11	2.4 [1.6–3.6]	< 0.0001
Marital status									
Single	0.31	reference	–	0.44	reference	–	0.03	reference	–
Married	0.33	1.03 [0.81–1.3]	0.7592	0.41	0.93 [0.74–1.2]	0.5013	0.07	2.1 [1.2–3.7]	0.0092
Widowed, divorced or separated	0.27	0.86 [0.59–1.3]	0.4404	0.38	0.87 [0.61–1.3]	0.4253	0.10	3.1 [1.6–6.2]	0.0013
Education									
Elementary	0.15	reference	–	0.28	reference	–	0.22	reference	–
Secondary	0.28	1.8 [0.94–3.5]	0.0736	0.38	1.3 [0.77–2.3]	0.3034	0.08	0.35 [0.19–0.66]	0.0011
College or university	0.35	2.3 [1.2–4.3]	0.0139	0.44	1.6 [0.92–2.7]	0.1002	0.05	0.20 [0.11–0.38]	< 0.0001
Employment									
Employed/self-employed	0.33	reference	–	0.43	reference	–	0.05	reference	–
Unemployed or housewife	0.26	0.81 [0.60–1.1]	0.1468	0.36	0.77 [0.59–1.0]	0.0632	0.10	1.8 [1.2–2.9]	0.0068
Student or retired	0.33	1.0 [0.81–1.3]	0.8508	0.42	0.96 [0.76–1.2]	0.7005	0.06	1.03 [0.64–1.6]	0.9166
Altitude (m)									
< 1000	0.34	reference	–	0.45	reference	–	0.05	reference	–
1000-2000	0.36	1.1 [0.80–1.4]	0.6747	0.41	0.93 [0.72–1.2]	0.6156	0.06	1.2 [0.69–2.1]	0.4920
> 2000	0.24	0.69 [0.47–1.0]	0.0603	0.33	0.73 [0.51–1.1]	0.0869	0.08	1.5 [0.78–3.0]	0.2111
Body weight (BMI)									
Underweight (< 18.5)	0.21	0.61 [0.31–1.2]	0.1713	0.50	1.3 [0.73–2.3]	0.3813	0.06	1.1 [0.33–3.6]	0.8984
Normal (18.5–24.9)	0.35	reference	–	0.39	reference	–	0.05	reference	–
Overweight (25.0–29.9)	0.31	0.89 [0.70–1.1]	0.3230	0.44	1.1 [0.92–1.4]	0.2284	0.07	1.4 [0.89–2.1]	0.1494
Obese (≥30)	0.28	0.80 [0.59–1.1]	0.1362	0.41	1.07 [0.81–1.4]	0.6348	0.09	1.8 [1.1–2.9]	0.0214

*pMOH* Probable medication-overuse headache, *OR* Odds ratio, *CI* 95% confidence interval.

We compared the urban dwellers of Ulaanbaatar (*n* = 817) with the rural participants from the four aimags (*n* = 1226). Migraine was less common among the latter (OR: 0.80; *p* = 0.0326). TTH showed no differences but, on the other hand, pMOH was considerably more common in rural areas (OR: 2.4; p < 0.0001) (Table 3).

We compared those who were single (*n* = 481) with those who were married (*n* = 1341) or widowed, divorced or separated (*n* = 221). Migraine showed no differences. TTH was less common in married females than single (OR: 0.75; *p* = 0.0497) but not in married males (OR: 0.93). However, pMOH was substantially more common among the other groups than among participants who were single (Table 3). For those married (OR: 2.1; *p* = 0.0092), this difference was greatly magnified in males (OR: 5.4; *p* = 0.0214) while losing significance in females (OR: 1.6; *p* = 0.1273).

We compared those who had only elementary education (*n* = 82) with those who had received secondary (*n* = 689) or college or university education (*n* = 1271). Migraine was more common in the last category (OR: 2.3; *p* = 0.0139), with a trend towards being more common in those with secondary education (OR: 1.8; *p* = 0.0736). Thus there was a consistent association between this disorder and increasing educational level. TTH showed no differences, but a consistent association was also shown, albeit in the opposite direction, between pMOH and educational level: OR = 0.35 (*p* = 0.0011) in those with secondary education and OR = 0.20 in those with college or university education (*p* < 0.0001).

We compared those who were employed (including self-employed) (*n* = 1182) with the unemployed (*n* = 350) and students or retired (*n* = 511). The last group showed no differences. Between the other two, only pMOH showed a clear difference, being much more common among unemployed people (10.1% versus 5.4%; OR: 1.8; *p* = 0.0068) (Table 3).

We looked at altitude of dwelling. There were no differences between low altitude (< 1000 m) and intermediate (1000–2000 m); above 2000 m, there was a trend towards less prevalent migraine (OR: 0.69; *p* = 0.0603) (Table 3).

Finally, we considered BMI, excluding those known to be pregnant (*n* = 31) from this analysis. Half (50.8%) of the sample were of normal weight, but 30.2% were overweight and 16.1% obese. Neither migraine nor TTH showed any clear relationship. On the other hand, pMOH was significantly associated with obesity (OR: 1.8; *p* = 0.0214), with a trend towards increased prevalence in those overweight (OR: 1.4; *p* = 0.1494) (Table 3).

## Discussion

This nationwide population-based survey in Mongolia found a crude 1-year prevalence of any headache of 66.1%. Migraine was substantially more common among females than males, with age- and gender-adjusted 1-year prevalence of 23.1%. TTH, with no gender difference, had an age- and gender-adjusted 1-year prevalence of 29.1%. Associations with age were not strong, but prevalences were least at 55–65 years. Headache on ≥15 days/month (age- and gender-adjusted prevalence 10.7%) was also more common in females than males. Over half of cases (5.7%) were pMOH, which showed a strong association with age, peaking in middle years with a 5-fold increase in prevalence.

There were a few other significant associations. Migraine showed a consistent association with educational level, while pMOH showed the reverse (more common in the less well educated), this perhaps being reflected in its higher prevalence among the unemployed. However, pMOH was also substantially more common among other groups than among participants who were single, especially in males. We do not have anything to say about these. Migraine was less common among rural participants than urban, while pMOH again showed the reverse, which is perhaps surprising for two reasons. First, pMOH prevalence tends to be driven by (inter alia) migraine prevalence, since the latter disorder is, in most cases, the antecedent of the former. Second, access to medication is usually easier in urban areas (although, contrariwise, access to health care is not, which may overdrive self-medication).

While headache disorders were common, the most noteworthy finding was the very high prevalence of headache on ≥15 days/month, corroborated by the high prevalence of headache yesterday (20.1%), which also showed a strong female association. Most was accounted for by pMOH, but clinical studies are very much needed in Mongolia (as they are in Russia [16, 28]: see below) to ascertain cause in the remaining 5% of adults with this disabling disorder.

The comparisons to make are with Mongolia’s neighbours, Russia and China, as we noted in the Introduction. Mongolian headache matches Russian headache very closely [16]: migraine 23.1% versus 20.8%, TTH 29.1% versus 30.8% and headache on ≥15 days/month 10.7% versus 10.5%. This study therefore nicely corroborates the Russian study. Accordingly, however, Mongolian headache is greatly at odds with headache in China, its southern neighbour (9.3%, 10.8% and 1.0% respectively [17]). As judged from other LTB studies, Russia is broadly in line with Georgia [18] and Lithuania [19], and, at least with regard to episodic headache (although there is regional variation), with India [29], Nepal [30], Pakistan [31], Ethiopia [32] and Zambia [33]. China, as noted earlier, appears to be matched by Japan [20] and Taiwan [21], although the 20-year-old studies in these countries used different methodologies and may not have included probable migraine. Nevertheless, these very far-Eastern countries apparently differ from those in all other regions. While genetic factors are commonly invoked in attempts to explain this, it seems more likely that cultural distinctions influence illness perception and reporting behaviour rather than headache prevalence. In Korea, an intermediate estimate of 17.5%, obtained more recently but again through different methodology, expressly included probable migraine [34].

We should comment on altitude. There were no differences between low and intermediate altitudes, while above 2000 m there was a trend towards less prevalent migraine. In Nepal, an LTB-supported study (using the same methodology) found a very strong relationship between migraine prevalence (and indices of severity) and altitude up to 2000 m [30, 35], which is not reflected here. Explanation comes to mind: we only had approximations of altitude of each household, and the spread was rather narrow – certainly not as wide as in Nepal [35]. Thus, “low altitude” was 800–900 m while “intermediate” was 1000–1600 m, with most households at 1300 m. Interestingly, nonetheless, there was a downturn in prevalence above 2000 m, as was found (and not well explained) in Nepal [35].

Finally, while neither migraine nor TTH showed any relationship, pMOH demonstrated its well-documented association with obesity. That obesity is a risk factor for headache chronicity developing from migraine has long been reported (eg, [36]).

There are messages for health policy-makers. Mongolia has a high prevalence of headache, and of frequent headache – well above global means [3] (although global means almost certainly remain underestimated [4]). There can be no doubt that headache in Mongolia imposes heavy ill-health and economic burdens (these will be reported later).

This study was nationwide and conducted according to published standardized methodology [14, 15], gathering a representative sample of adults with a very high participation proportion (98.3%). Data collection was completed quickly, over 4 months. These are the study’s strengths. One limitation was the lack of validation of the diagnostic questions in Mongolian language: instead, we relied upon the earlier usage of these questions in > 20 languages, countries and cultures [14]. This limitation was enforced by the lack of headache specialists in Mongolia (a situation that this study may help to change). A second, somewhat troublesome, was evidence of biased sampling, despite much effort to minimise this by multistage randomisation. The gender bias has been explained (see Methods), but the apparent bias in educational level is more problematic: the sampling seems to have missed people with low education (4.0% against expected 28.4%) while over-sampling those with university education (48.0% against expected 19.8%). Two partial explanations offer themselves. First is the gender bias: at each higher level of education in Mongolia, there is an increasing female preponderance (two-fold in universities) [37, 38]. Second, there is uncertainty about the official statistics, which are from 2010, 8 years before our survey and at a time of rapid and ongoing reform in Mongolian education, designed to change from a highly specialised and compartmentalised system of education based on the Russian model to one that is more rational, flexible and decentralised [38]. Mongolia distinguishes between postsecondary and higher education on the one hand and technical education and vocational training (TEVT) on the other, the former largely but not entirely delivered by universities, while nonformal and distance education activities cut across the entire educational system [38]. Other apparently authoritative data sources are not in agreement with the official data. One, from 2000, reported 11.6% with TEVT and 35% with higher education [37] – much closer to our ratios. A WHO survey from 2006 reported 33.4% of those aged 15–64 years having completed secondary school, vocational training and college/university, females (38.1%) more than males (29.1%) [39]. This same survey reported only 11.8% with elementary (incomplete or complete primary) education. The problem may lie at the boundaries between levels, and how those who have partially completed a level report themselves on the one hand or might objectively be categorised on the other. In these circumstances, we are not certain what is the truth, or what corrections, if any, should be made.

## Conclusion

The prevalence of headache disorders among the adult population of Mongolia is high, with a very similar picture to neighbouring Russia and in excess of global means. Headache on ≥15 days/month is very common, as is pMOH, a main contributor. These disorders require special attention. This new evidence adds to knowledge of the global prevalence of headache. With analyses of headache-attributed burden, which will follow, it will also inform national health policy and provide a basis for the health-care needs assessment.

## Data Availability

The data are held on file at Mongolian National University of Medical Sciences and at Norwegian University of Science and Technology. Once analysis and publications are completed, they will be freely available for non-commercial purposes to any person requesting access in accordance with the policy of the Global Campaign against Headache.
